# Distribution and phylogeny of *Wolbachia* strains in wild mosquito populations in Sri Lanka

**DOI:** 10.1186/s13071-017-2174-9

**Published:** 2017-05-10

**Authors:** N. W. Nalaka P. Nugapola, W. A. Priyanka P. De Silva, S.H.P. Parakrama Karunaratne

**Affiliations:** 1 Department of Zoology, University of Peradeniya, Peradeniya, 20400 Sri Lanka; 2 Postgraduate Institute of Science, University of Peradeniya, Peradeniya, 20400 Sri Lanka; 3 National Institute of Fundamental Studies, Hantana Road, Kandy, 20000 Sri Lanka

**Keywords:** *Wolbachia* strains, Phylogeny, *Wsp*, Biological control, Mosquito control, Sri Lanka

## Abstract

**Background:**

*Wolbachia* are a group of maternally inherited intracellular bacteria known to be widespread among arthropods. Infections with *Wolbachia* cause declines of host populations, and also induce host resistance to a wide range of pathogens. Over the past few decades, researchers were curious to use *Wolbachia* as a biological tool to control mosquito vectors. During the present study, assessment of the prevalence of *Wolbachia* infections among wild mosquito populations in Sri Lanka where mosquito-borne diseases are a major health concern, was carried out for the first time. DNA was extracted from the abdomens of mosquitoes, collected from seven provinces, and screened for the presence of *Wolbachia* by PCR using *wsp* and *groE* primers. Group-specific and strain-specific primers were used to classify *Wolbachia* into the supergroups A and B, and into the strains *Mel*, *AlbA* and *Pip.*

**Results:**

A total of 330 individual mosquitoes belonging to 22 species and 7 genera were screened. Eighty-seven mosquitoes (26.36%) belonging to four species (i.e. *Aedes albopictus*, *Culex quinquefasciatus*, *Armigeres subalbatus* and *Mansonia uniformis*) were positive for *Wolbachia* infections. Primary vector of the dengue fever, *Ae. aegypti* was negative for *Wolbachia* infections while the secondary vector, *Ae. albopictus*, showed a very high infection rate. The filarial vector *C. quinquefasciatus* had a relatively high rate of infection. Japanese encephalitis vectors *C. gelidus* and *C. triteaneorynchus*, and the *Anopheles* vectors of malaria were negative for *Wolbachia* infections. Nine sequences of *Wolbachia*-positive PCR products were deposited in the GenBank and compared with other available data. *Aedes albopictus* was infected with both *Wolbachia* strains A (*AlbA*) and B (*Pip*) supergroups. Phylogenetic analysis of the *wsp* sequences showed two major branches confirming identities obtained from the PCR screening with strain-specific primers.

**Conclusion:**

*Wolbachia* infections were found only among four mosquito species in Sri Lanka: *Aedes albopictus*, *Culex quinquefasciatus*, *Armigeres subalbatus* and *Mansonia uniformis*. Sequence data showed high haplotype diversity among the *Wolbachia* strains.

## Background

Environment-friendly cost-effective strategies have emerged as crucial approaches in vector control programs today. The potential application of the symbiotic *Wolbachia* bacteria as a novel form of biological tool to control of mosquito-borne diseases, particularly dengue, has attracted an enormous attention during the past few decades. *Wolbachia* is a group of maternally inherited intracellular bacteria which shows symbiotic relationships with arthropods and nematodes [1, 2]. They were first discovered by Hertig & Wolbach [3] and since then the biology, diversity, distribution, evolution and the rate of host infection of *Wolbachia* have been studied widely [1, 2, 4–7].

Infection with *Wolbachia* results in various reproductive abnormalities in the host including feminization, parthenogenesis, male killing and cytoplasmic incompatibility (CI) [1]. High infection rates, caused mainly through reproductive manipulations of the host, have enabled *Wolbachia* to spread rapidly within host populations making them a worthy candidate tool for consideration in vector control programs. Use of *Wolbachia* induced CI as a tool for mosquito control had been proposed in as early as 1960s [8] and few trials had been conducted in India in the 1970s [9]. Development of anti-*Wolbachia* based therapies to control mosquito borne diseases by replacement of vector populations and CI based incompatible insect techniques (IIT) are some of the recent approaches [10–13]. Successful control of the dengue vector mosquito *Aedes aegypti* by the introduction of *Ae. aegypti* infected with laboratory developed *wMel* and *wMelPop* strains of *Wolbachia*, to natural mosquito populations has been reported from the field trials conducted in Australia, Vietnam, Indonesia, Brazil and Colombia [14, 15]. Interestingly, the presence of *Wolbachia* induces host resistance to a wide range of pathogens including viruses, bacteria, protozoans and nematodes in trans-infected mosquitoes [13, 16–18]. Increased host resistance to pathogens is thought to be mainly through enhancing host immune responses, including haemolymph melanization, by *Wolbachia* and also through direct competition for cellular resources [13].

*Wolbachia* strains are globally distributed and currently considered as the most abundant endosymbionts found in invertebrates [19]. About 40% of arthropod species are estimated to be infected with *Wolbachia* [20]. In arthropods, *Wolbachia* are believed to be primarily maternally transmitted within species [21] although horizontal transmission is also possible [22, 23]. Previous studies based on 16S rRNA gene sequences reported seven super-groups (super-groups A-H) of *Wolbachia* [2, 4]; supergroups A and B are the most common *Wolbachia* reported in arthropods [2]. Zhou et al. [5] reported that an improved phylogenetic resolution can be achieved by using the sequences of *wsp* gene which is evolving at a much faster rate than the other *Wolbachia* genes.

To our knowledge, the presence of *Wolbachia* in natural populations of mosquitoes has never been studied in Sri Lanka where mosquito-borne diseases are a major public health concern and more than 50,000 dengue cases have been reported among its population of 21 million people for the year 2016 [24]. Mosquito control programs of the country heavily rely on fogging operations using chemical insecticides [25] despite the increased vector resistance to insecticides and the adverse effect of insecticide fogging on non-target insects [26]. Environment-friendly new alternatives are urgently needed for vector control programs. The present study was undertaken to investigate the prevalence of *Wolbachia* infections among wild mosquito populations from different geographical regions of the country. Phylogenetic analysis of *wsp* sequences of the identified *Wolbachia* strains was also performed.

## Methods

### Mosquito collection and identification

**Fig. 1:**
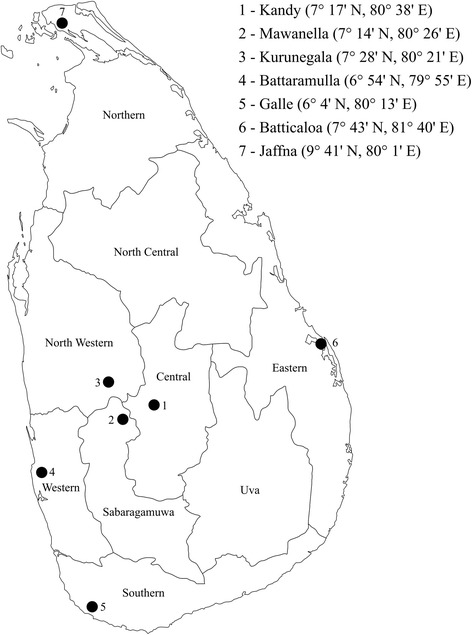
A map of Sri Lanka showing the provinces and the collection sites

Adults, larvae and eggs of different mosquito species were collected from seven provinces (i.e. Central, Sabaragamuwa, Western, Southern, Northern, North-western and Eastern) of Sri Lanka (Fig. 1). Adult collections were done using BG-Sentinel traps and CDC light traps. Larvae were collected using dippers and pipettes from mosquito breeding places. Mosquito eggs were collected using ovitraps. Larvae and eggs were reared to adults under laboratory conditions. Adult mosquitoes were identified using the standard taxonomic keys [27–30]. Identified specimens were labeled and stored at -20 °C for later use.

### DNA extraction and polymerase chain reaction (PCR)

DNA from the mosquito abdomens were extracted individually following the method described by Livak [31]. Five microliters from the extract was used for the PCR and double distilled water was used as the negative control. A known extract of *Wolbachia*-infected *Ae. albopictus* sample was used as the positive control.

Presence of *Wolbachia* was tested by amplifying *Wolbachia* surface protein gene using general *wsp* primers [5, 7, 32], i.e. *wsp*81F (5′-TGG TCC AAT AAG TGA TGA AGA AAC-3′) and *wsp*691R (5′-AAA AAT TAA ACG CTA CTC CA-3′). Resulting DNA fragments ranged from 590 to 632 bp depending on the *Wolbachia* strain. PCR amplifications were done in a thermal cycler (Techne-Flexigene, England) in 25 μl reaction volumes. Each 25 μl PCR reaction contained 7.5 μl GoTaq^®^ Green Master Mix (Promega, USA), 1.0 μl each from forward and reverse primers (Macrogen, Korea), 5 μl DNA templates and 10.5 μl nuclease free water (Promega, USA). The PCR cycling conditions were: initial denaturing at 95 °C for 3 min, followed by 30 cycles of 94 °C for 1 min, 55 °C for 1 min and 72 °C for 1 min. The final elongation was done at 72 °C for 10 min, while the final holding was done at 4 °C.

*GroE* primers, which amplify the 630 bp region from the *groE* operon [33], were also used to check for the presence of *Wolbachia*. PCR mixture composition was same as for *wsp* primers and the PCR cycling conditions were: initial denaturing at 95 °C for 3 min, followed by 30 cycles of 94 °C for 1 min, 48 °C for 1 min and 72 °C for 1 min, final elongation at 72 °C for 10 min and final holding at 4 °C.

### Identification of *Wolbachia* groups and strains

Samples positive for the *wsp* primers were subjected to *Wolbachia* strain- and group-typing with *wsp* specific primers [5]. Group-specific primers were used to classify *Wolbachia* into supergroups A and B. Samples that were positive for supergroup A were screened with the *Wolbachia* strain-specific primers *Mel* and *AlbA. S*upergroup B samples were screened with *Pip* primer. Same PCR parameters described above were used. Five microliters of the PCR products were run on 1.5% agarose gel with a 100 bp standard DNA size marker (Invitrogen, California, USA) to confirm the PCR amplification. Samples with a DNA band of expected size were scored as positive.

### *Wsp* sequencing and phylogenetic analysis

Nine *Wolbachia*-positive PCR products were directly sequenced using an automatic DNA sequencer (Applied Biosystems series 3500, USA). Sequence data were compared with the available data in the GenBank database using BLAST search and the sequences were deposited in the GenBank under accession numbers KY523666–KY523674.

*Wsp* sequences obtained from this study, reference *wsp* sequences obtained from EMBL alignment database [34] (accession no. DS32273) and *wsp* sequences obtained from GenBank [35] (KJ140127, AF020058, KF725078, KF725079, KC668277, KC668275, KF725080, KC668284, KC668278, KJ140133, AY462864, GQ469982, KC137171, HM007832, DQ842453, HM007831) were edited and aligned in BioEdit 7.2.5 [36] using CustalW alignment algorithm [37]. Evolutionary history was inferred using the neighbor-joining (NJ) method [38] and the evolutionary distances were computed using the Kimura 2-parameter method [39]. Evolutionary analyses were conducted in MEGA7 [40].

## Results

A total of 330 individual mosquitoes belonging to 22 species and 7 genera, collected from the 7 provinces of Sri Lanka, were individually screened for the presence of *Wolbachia* by PCR using *wsp* and *groE* primers. Presence of *Wolbachia* in different mosquito species from each province is given in the Table 1. A total of 87 mosquitoes, out of 330 (26.36%), were positive for *Wolbachia* infection. Out of 22 species screened only 4 species, i.e. *Ae. albopictus*, *Culex quinquefasciatus*, *Armigeres subalbatus* and *Mansonia uniformis*, were infected.

**Table 1:** Prevalence of *Wolbachia* in mosquito species collected from different provinces of Sri Lanka

Mosquito species	Province							Total	% infected
	C	W	E	N	NW	SG	S		
*Aedes aegypti*	0/17	0/2	0/1	0/10	0/10	–	–	0/40	0.0
*Aedes albopictus*	33/33	1/29	–	–	9/29	10/10	9/26	62/127	48.8
*Aedes vittatus*	–	–	–	–	0/2	–	–	0/2	0.0
*Anopheles* species^a^	0/1	–	0/35	–	0/46	–	–	0/82	0.0
*Armigeres subalbatus*	4/4	–	–	–		–	3/3	7/7	100.0
*Culex annulirostris*	–	–	–	–	–	–	0/2	0/2	0.0
*Culex gelidus*	–	–	0/4	–	–	–	0/2	0/6	0.0
*Culex mimulus*	–	–	–	–	0/5	–	–	0/5	0.0
*Culex quinquefasciatus*	5/17	–	0/13	–	8/17	–	2/3	15/50	30.0
*Culex triteaneorynchus*	–	–	0/2	–	–	–	0/1	0/3	0.0
*Mansonia uniformis*	–	–	–	–	–	–	3/3	3/3	100.0
*Mimomia elegans*	0/1	–	–	–	–	–	–	0/1	0.0
*Uranotaenia rutherfordi*	0/2	–	–	–	–	–	–	0/2	0.0
Total	42/75	1/31	0/55	0/10	17/109	10/10	17/40	87/330	26.36

*Abbreviations*: *C* Central, *W* Western, *S* Southern, *N* Northern, *E* Eastern, *SG* Sabaragamuwa, *NW North-Western*

^a^ *Anopheles* species: *Anopheles barbirostris* (*n* = 10), *An. jamesii* (*n* = 13), *An. karwari* (*n* = 4), *An. pallidus* (*n* = 2), *An. peditaeniatus* (*n* = 15), *An. subpictus* (*n* = 18), *An. tessellatus* (*n* = 6), *An. varuna* (*n* = 7), *An. vegus* (*n* = 7)

The primary vector of the dengue fever *Ae. aegypti* was negative (*n* = 40) for *Wolbachia* infection. The secondary vector of the dengue fever, *Ae. albopictus*, showed a 100% infection rate for the mosquitoes collected from Central (*n* = 33) and Sabaragamuwa (*n* = 10) provinces followed by Southern (34.6%; 9 out of 26) and North Western provinces (31.0%; 9 out of 29). Only one *Ae. albopictus* was positive for *Wolbachia* from the Western province Pamples (3.4%; 1 out of 29). *Wolbachia* infection rate was relatively high in the filarial vector *C. quinquefasciatus*; infection rates in this species from Southern, North-Western and Central provinces were 66.7, 47.1 and 29.4%, respectively. However, *C. quinquefasciatus* collected from the Eastern Province (*n* = 13) were negative for *Wolbachia* infections. The Japanese encephalitis vectors *C. gelidus* (*n* = 6) and *C. triteaneorynchus* (*n* = 3) were not infected with *Wolbachia.* All of the *Anopheles* species tested (*n* = 82) were also negative for the *Wolbachia* infections. Infection rate of *M. uniformis* and *Ar. subalbatus* were 100% (*n* = 3 and *n* = 7, respectively).

**Fig. 2:**
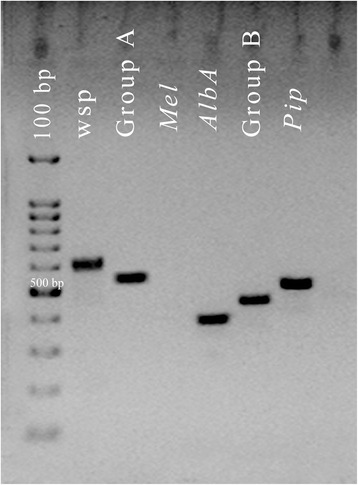
Results of the *Wolbachia* strain identification PCR assays for *Aedes albopictus* mosquitoes collected from the central province of Sri Lanka (*n* = 33), using different primer sets: Lane 1: *Wolbachia* confirmation general primers *wsp*81F and *wsp*691R (600 bp band); Lane 2: Group A primers wsp136 and wsp691R (556 bp); Lane 3: *Mel* strain-specific primers *wsp*308F and *wsp*691R (405 bp); Lane 4: *AlbA* strain-specific primers wsp328F and 691R (379 bp); Lane 5: Group B primers wsp81F and wsp522R (442 bp); Lane 6: *Pip* strain-specific primers wspp183F and wsp 691R (501 bp). PCR products were electrophoresed in 1.5% agarose gel, stained with ethidium bromide and visualized under UV light

Results of the PCR performed with *Wolbachia* group-specific *wsp* primers showed that *Ae. albopictus* (*n* = 10) is super-infected with *Wolbachia* strains belonging to A and B supergroups. Further screening using strain-specific *wsp* primers revealed that the strain of *Wolbachia* in supergroup A is *AlbA* (379 bp band in agarose gel) and the strain in supergroup B is *Pip* (501 bp band in agarose gel) (Fig. 2). *Culex quinquefasciatus* (*n* = 5) and *M. uniformis* (1 out of 2) were infected with *Pip* strain of *Wolbachia* which belongs to the supergroup B and *Ar. subalbatus* (2 out of 3) was infected with *AlbA* strain (Table 2).

**Table 2:** Different groups and strains of *Wolbachia* present in the mosquito species *Aedes albopictus* (*n* = 10), *Culex quinquefasciatus* (*n* = 5), *Armigerus subalbatus* (*n* = 4) and *Mansonia uniformis* (*n* = 2)

*Wolbachia* super-group	Strain	Primers (5′-3′)	PCR product (bp)	AA^a^	CQ^a^	AR^a^	MN^b^
Group A		136 F: TGAAATTTTAGCTCTTTTC 691R: AAAAATTAAACGCTACTCCA	556	10	–	03	–
	*Mel*	308 F: TTAAAGATGTAACATTTG 691R: AAAAATTAAACGCTACTCCA	405	–	–	–	–
	*AlbA*	328 F: CCAGCAGATACTATTGCG 691R: AAAAATTAAACGCTACTCCA	379	10	–	02	–
Group B		81 F: TGGTCCAATAAGTGATGAAGAAAC 522R: ACCAGCTTTTGCTTGATA	442	10	05	–	02
	*Pip*	183 F: AAGGAACCGAAGTTCATG 691R: AAAAATTAAACGCTACTCCA	501	10	05	–	01

^a^From Central Province

^b^From Southern Province

*Abbreviations*: *AA* *Aedes albopictus*, *CQ Culex quinquefasciatus*, *AR Armigerus subalbatus*, *MN Mansonia uniformis*

**Fig. 3:**
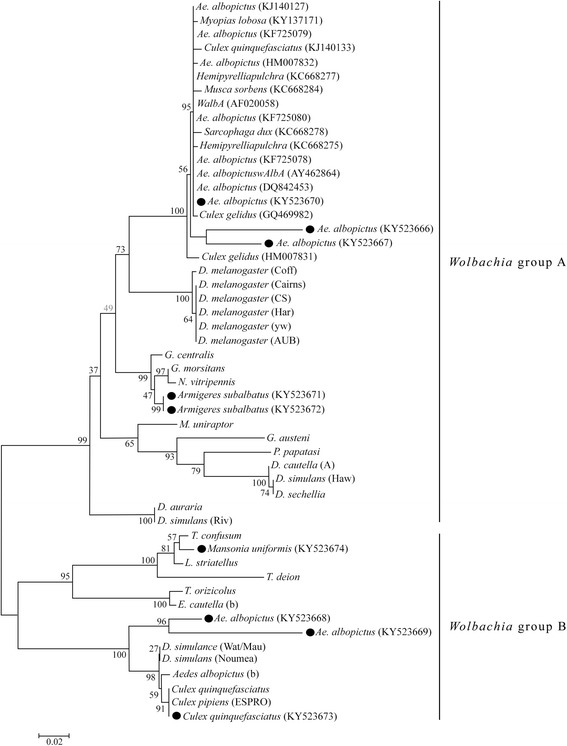
Neighbor-joining tree generated from aligned *wsp* sequences. Tree shown is midpoint rooted and bootstrap values (1,000 replicates) are labelled next to the branches. Taxa are labelled as the host names from which the *Wolbachia* strains were obtained. *Wsp* sequences from the present study are marked with black dots

The results of the phylogenetic analysis of *Wolbachia* using *wsp* sequences obtained from the present study and other 43 reference sequences are shown in Fig. 3. NJ tree was rooted between A and B super groups, and bootstrap values showed strong support for this separation. The phylogeny based on *wsp* sequences clearly showed that *Wolbachia* strains harbored by *Ae. albopictus* separated into A and B supergroups with strong bootstrap values. Of five *Wolbachia* sequences obtained from *Ae. albopictus*, three (KY523666, KY523667 and KY523670) were in super group A and two (KY523668 and KY523669) were in super group B (Fig. 3). *Wolbachia* present in *Ar. subalbatus* (KY523671 and KY523672) were also clustered into *Wolbachia* supergroup A, but in a separate clade from *Wolbachia* present in *Ae. albopictus* mosquitoes. *Wolbachia* harbored by *C. quinquefasciatus* (KY523673) and *M. uniformis* (KY523674) were separated into *Wolbachia* supergroup B.

## Discussion

To our knowledge, this is the first report to show the presence of *Wolbachia* bacteria in natural mosquito populations in Sri Lanka. The result of the PCR assays confirmed the previous findings that both *wsp* and *groE* primers are capable of identifying *Wolbachia* infections [7]. According to our results, the overall infection rate of all tested mosquitoes was 26.36%. For natural mosquito populations, *Wolbachia* infection rates of 28.1 and 37.8% have been previously reported in Singapore [32] and Thailand [7], respectively.

In Sri Lanka, *An. culicifacies* is the major vector of malaria and *An. subpictus* is considered as a secondary vector. All of the other anopheline species tested have been incriminated as possible vectors of malaria [41]. *Wolbachia* could not be detected in any of the *Anopheles* species screened and this observation is consistent with previous reports [7, 32, 42]. *Wolbachia*-infected *C. quinquefasciatus* has been reported previously [43, 44]. *Wolbachia* was absent in the tested samples of the vectors of Japanese encephalitis, *C. tritaeniorhynchus* and *C. gelidus*; however, Wiwatanaratnabutr [7] had earlier encountered *Wolbachia* infections in both vectors. Kittayapong et al. [32] has reported that *C. tritaeniorhynchus* was positive for *Wolbachia* while *C. gelidus* was negative. The absence of *Wolbachia* infections in these vectors in Sri Lanka may be due to the small numbers of mosquitoes tested.

Although natural *Wolbachia* infections have never been reported from populations of the primary dengue vector *Ae. aegypti*, high infection rates have always been reported for *Ae. albopictus* mosquitoes [7, 15, 45]. For some reason infection rate in *Ae. albopictus* was remarkably low in the Western Province, i.e. one out of 29. During the present study, supergroup-typing of *Wolbachia* using PCR showed that all *Ae. albopictus* samples tested were co-infected with both A and B groups of *Wolbachia*. Specific primers derived from *Wolbachia* surface protein gene (*wsp*) have been used for *Wolbachia* strain identification and phylogeny analysis previously and were said to be the most sensitive for such an analysis [5]. The AB double infection of *Wolbachia* in *Ae. albopictus* at a rate of 99.4% has been previously reported from Thailand [32, 45]. It has been reported that multiple infections with several *Wolbachia* groups induce cytoplasmic incompatible phenotypes in female mosquitoes [1]. Further investigation of *Wolbachia* groups into strains revealed that all *Ae. albopictus* samples tested were commonly infected with *AlbA* (group A) and *Pip* (group B) strains of *Wolbachia.* Kittayapong et al. [45] reported the same combination of strains of *Wolbachia* (*AlbA* and *Pip*) in *Ae. albopictus*, *Ae. pseudoalbopictus* and other *Ae.* (*Stegomyia*) spp. They reported that three out of five species of the subgenus *Stegomyia* were infected with both *AlbA* and *Pip* strains of *Wolbachia.* Our results also revealed the presence of the strain *Pip* in *C. quinquefasciatus* and *M. uniformis*, and the strain *AlbA* in *Ar. subalbatus*.

The identities obtained from the PCR screening with strain-specific primers were further confirmed during the phylogenetic analysis of the *wsp* sequences. It is evident that haplotype diversity of *Wolbachia* strains was very high. According to the NJ tree generated, three *wsp* sequences (*Ae. albopictus*: KY523666, KY523667 and KY523670) were clustered with a clade where *Wolbachia AlbA* sequences (AF020058 and AY462864) clustered together. Other sequences (*Ae. albopictus*: KY523668, KY523669; *Culex quinquefasciatus*: KY523673; *Mansonia uniformis*: KY523674) were in the Group B branch of the phylogenetic tree where *wsp* sequences of *Wolbachia Pip* strains from *Cx. pipiens*, *Cx. quinquefasciatus, Drosophila simulans* and *Ae. albopictus* (b) are found (Fig. 2).

Incidence of dengue is increasing at an alarming rate in Sri Lanka and other tropical countries. Control of dengue vector larvae with the common strategies such as spraying synthetic insecticides/*Bacillus thuringiensis* is not effective as the breeding sites of dengue vectors are mainly discarded receptacles and leaf axels among the vegetation [46]. Although the clearance of breeding sites is effective to a certain extent, elimination of vegetation is not possible. Efficacy of space spraying of adulticides is also questionable considering the actual effect on the mosquitoes and the extent of damage it causes to the non-target insects [26]. There is an urgent need to adopt alternative techniques such as *Wolbachia*-infected replacement [11–13], sterile male technique and radiation to combat the dengue and other vector populations in endemic areas [47–50]. The widespread distribution and various reproductive manipulations of *Wolbachia* have drawn attention of researchers to use *Wolbachia* as a biological control method. This approach is attempted by many researchers around the world and the field trials have demonstrated promising outcomes [10, 13–15].

Given the fact that *Wolbachia* reduces the life span of the host preventing the virus to complete its proper incubation period and also causes increased host immunity resisting the viral development in the host, *Wolbachia* would be a better choice for vector control leading to a large-scale reduction of the disease transmission. The present study is an initial attempt to produce basic information regarding the extent of natural infections of *Wolbachia* in wild mosquito populations in Sri Lanka. Although it is not found among wild *Ae. aegypti* populations, trans-infected *Ae. aegypti* has been successfully used in field trials [14, 15, 51–53]. Trans-infected mosquito lines may also be developed to combat diseases such as malaria and Japanese encephalitis. The present study, therefore provides important information to develop novel approaches in using *Wolbachia* to mosquito control, particularly in controlling mosquito populations that do not respond to conventional vector control strategies.

## Conclusions

*Wolbachia* infections were found only among four mosquito species in Sri Lanka: *Aedes albopictus*, *Culex quinquefasciatus*, *Armigeres subalbatus* and *Mansonia uniformis*. Sequence data showed high haplotype diversity among the *Wolbachia* strains. *Ae. albopictus* samples were commonly infected with *AlbA* (group A) and *Pip* (group B). *Pip* was also found in *C. quinquefasciatus* and *M. uniformis. Wolbachia* strain *AlbA* was found in *Ar. subalbatus*.
